# Sulfur Amino Acids in Diet-induced Fatty Liver: A New Perspective Based on Recent Findings

**DOI:** 10.3390/molecules19068334

**Published:** 2014-06-19

**Authors:** John I. Toohey

**Affiliations:** Cytoregulation Research, Elgin, ON K0G 1E0, Canada; E-Mail: cytoreg@xplornet.com

**Keywords:** fatty liver, hepatosteatosis, lipotropes, sulfane sulfur, sodium hydrosulfide, methionine, homocysteine, mercaptoethanol, cystamine, cystine, garlic sulfur compounds

## Abstract

The relationship of sulfur amino acids to diet-induced fatty liver was established 80 years ago, with cystine promoting the condition and methionine preventing it. This relationship has renewed importance today because diet-induced fatty liver is relevant to the current epidemics of obesity, non-alcoholic fatty liver disease, metabolic syndrome, and type 2 diabetes. Two recent papers provide the first evidence linking sulfane sulfur to diet-induced fatty liver opening a new perspective on the problem. This review summarizes the early data on sulfur amino acids in fatty liver and correlates that data with current knowledge of sulfur metabolism. Evidence is reviewed showing that the lipotropic effect of methionine may be mediated by sulfane sulfur and that the hepatosteatogenic effect of cystine may be related to the removal of sulfane sulfur by cysteine catabolites. Possible preventive and therapeutic strategies are discussed.

## 1. Sulfur Amino Acids in Diet-Induced Fatty Liver: Early Findings

Diet-induced fatty liver was studied intensively following the discovery of insulin when it was observed that the depancreatized dogs developed this condition. Early data showed a close relationship of sulfur-containing amino acids to the causation or prevention of fatty liver. Since then, there have been many developments in the field of sulfur metabolism. To bring the two subjects together, it is necessary to review some of the old data on fatty liver and some modern sulfur biochemistry. Two recent papers throw new light on the subject.

### 1.1. Diets Inducing Fatty Liver

The normal fat content of mammalian liver is about 5% and it is increased to as much as 40% when rats or mice are fed hepatosteatogenic diets for 18 to 21 days. Several types of diets which induce fatty liver are listed below. Sucrose is used as the variable component and the diets are balanced by adding salt and vitamin mixes (omitted in the description of diets here).

A. Methionine-choline-deficient diets originally contained 5% casein which has been repeatedly extracted with boiling ethanol to remove choline. (Casein contains 0.34% cystine and 2.5% to 3.0% methionine.) Today these diets are made with purified amino-acids.

B. So called “fat” fatty liver was induced by diets containing 5% choline-free protein and 20% to 40% lard or beef fat.

C. So-called “cholesterol” fatty liver. Normal diets containing 15% fat or 1% to 2% cholesterol have little if any effect on liver fat but, when the two are combined, they act synergistically to cause fatty liver with both the sterol and the glyceride fractions greatly increased [1].

D. High cystine diets. Cystine is considered to be non-essential since it is derived from methionine. The diet recommended for rodents by the American Institute of Nutrition (AIN-76) contains 0.06% cystine [Zeiglerfeeds.com/products]. Adding cystine at 0.1% or greater to methionine-choline-deficient or high fat diets greatly increases the hepatosteatogenic properties of the diets.

E. Vitamin B_12_ deficiency. In the original description of pernicious anemia in 1855, Addison described “fatty degeneration of the organs”. Fatty liver has been demonstrated experimentally in chick [2] and rat [3] embryos from B_12_-deficient mothers. In cobalt-deficient parts of Australia, deficiency of Vitamin B_12_ causes severe fatty liver in sheep, called “ovine white liver disease” [4].

Other dietary factors which promote fat accumulation in the liver under some conditions include: high levels of sucrose, glucose, fructose, glycerol, thiamin, and biotin (all factors associated with lipid biosynthesis and reflecting the normal function of the liver in storing calories during periods of food abundance), starvation, Vitamin B_6_ deficiency, deficiency of certain essential amino acids particularly threonine and lysine (this deficiency causes increased food intake which promotes fatty liver), and deficiency of essential fatty acids [5].

### 1.2. Lipotropic Factors, Methionine and Choline

Early on, it was recognized that increasing the % casein in the diet decreased the amount of fat in the liver. Representative data are shown in Table 1. (All cited data are averages for 10 animals and the variability, which was within ±10%, is omitted for brevity.) Studies with separate amino acids identified the effective component of protein as methionine (Table 2).

**Table 1:** Effect of protein, casein (from [6]).

Diet	Casein (% of Diet)	Liver % Fat
Fat 20% Cholesterol 2% Casein varied	5	32.1
	10	26.4
	15	17.3
	20	14.8

**Table 2:** Effect of Methionine (from [7]).

Diet	Methionine Added	Liver % Fat
Egg albumin 5%	0	23.6
Fat 40%	0.25%	14.0
Egg albumin 5%	0	33.8
Fat 30%	0.25%	19.1
Cholesterol 2%		

Similarly, including lecithin in the diet reduced the amount of fat in the liver and the effective component was identified as choline, (CH_3_)_3_N^+^-CH_2_-CH_2_-OH. In the early experiments, choline was added to diets at 0.08% to 0.15%. More refined data generated by Best at al. in 1950 for varied choline are shown in Table 3. In the absence of excess cystine, choline produced maximum effect at about 0.03% of the diet. Choline has been considered to act by forming the phosphatidyl derivative (lecithin) which facilitates export of fat from the liver although this long-standing theory has been recently challenged [8].

**Table 3:** Effect of Choline (from [9] and [10]).

Diet	Choline	Diet A no Cystine	Diet B + Cystine
	% of Diet	% Fat in Liver	
Diet A: Casein 8% Gelatin 12%	0	15.1	34.5
	0.01	12.3	
	0.02	9.8	34.5
	0.04	6.0	27.6
Diet B: Protein 12% Fat 15% Cystine 0.2%	0.06	6.0	13.7
	0.08		8.9
	0.16	6.0	6.2
	0.32		5.2

The lipotropic effect of methionine has been attributed to its role in providing methyl groups via S-adenosylmethionine to convert phosphatidylethanolamine to phosphatidylcholine. However, there are facts which contradict this mechanism. Firstly, the human genetic defect in methionine-ATP adenosyl transferase causes acute deficiency of transmethylation capacity in the liver but fatty liver is not associated with that condition. Conversely, in human hyperhomocysteinemia (HHE) due to defective cystathionine beta synthase (CBS), methionine and S-adenosylmethionine are greatly increased but fatty liver occurs in most of these cases (although it is argued that the elevated S-adenosyl homocysteine inhibits the necessary transmethylations). Thirdly, choline and methionine appear to act on different components of liver fat. When the two were compared in “cholesterol” fatty livers and the liver fat was fractionated (Table 4), methionine (as protein, methionine % of diet shown in brackets) restored the glyceride fraction to normal but caused a consistent graded increase in the cholesterol ester while additional choline had a complete corrective effect on glycerides and partial corrective effect on cholesterol esters.

**Table 4:** Effect of Methionine on Lipid Fractions (from [6]).

Diet	Casein (Met)	Glyceride % of Liver		Cholesterol Ester % of Liver	
	% of Diet	Control	Choline 0.75%	Control	Choline 0.75%
Fat 20% Cholesterol 2% Choline ±0.75%	5 (0.12)	24.8	0.98	5.16	3.42
	10 (0.25)	16.2	3.40	7.15	4.66
	15 (0.36)	6.1	2.26	7.43	4.57
	30 (0.75)	2.9	2.71	7.72	6.45

### 1.3. Cystine

In 1935, cystine was tested with the expectation that it would replace methionine in its lipotropic effect but, to the surprise of the researchers, cystine caused a marked increase in the triglycerides in the livers (Table 5).

**Table 5:** Effect of Cystine (from [7]).

Diet		Glyceride % of Liver	Cholesterol % of Liver	Cholesterol Ester % of Liver
Casein 5%	Control	13.2	0.29	3.20
Beef fat 20%	Cystine 0.2%	32.7	0.26	3.35
Cholesterol 2%				

The livers in Table 5 already had a high fat level because the basal diet was hepatosteatogenic. Choline could prevent the cystine-induced increase in liver fat. In the system cited in Table 3, 0.03% choline prevented fatty liver when no cystine was added but 0.12% choline was needed when cystine was added at 0.2%. Homocystine was compared with cystine for its effect on fatty liver in diets containing 5% protein and 40% fat and found to be equally effective as cystine in causing fatty liver in rats [11] and mice [12]. However, the mechanisms may be different as discussed below.

The opposing effects of cystine and methionine prompted an analysis of the cystine/methionine ratio. The data in Table 6 were obtained by Treadwell *et al.* on varying cystine while methionine was held constant at 0.62% and varying methionine while cystine was held constant at 0.62% in a severely choline-deficient diet.

**Table 6:** Cystine/Methionine Ratio (from [13]).

Diet	Cystine Varied (met 0.62%)		Methionine Varied (cystine 0.62%)	
	Cys %	% Fat in Liver	Met %	% Fat in Liver
Casein 5% Agar 2% Lard 40% Choline 0	0.117	11.6	0.155	35.1
	0.217	11.2	0.310	32.8
	0.417	14.0	0.465	19.0
	0.617	12.0	0.775	9.9
	0.817	15.7		
	1.017	19.7		

The data show that it takes 0.62% methionine to neutralize the lipogenic effect of 0.62% cystine; *i.e.*, methionine and cystine have counteractive effects at a roughly equal amounts. When choline is also present in the diet, the following generalization can be drawn from scores of published data sets: [cystine] ≡ [methionine] + [choline] × 2. That is, the effect of an amount of cystine is counteracted by the sum of the amount of methionine plus the amount of choline multiplied by 2. This relationship can be used to predict whether any diet will be hepatosteatogenic; thus, if the value on the left is greater, the diet will cause fatty liver and vice versa.

To summarize the above, by 1950 research on diet-induced fatty liver had identified several causative and corrective factors and established the optimal concentrations for their effects as follows: lipogenic factors: fat, 15% to 40%; cholesterol, 2%; cystine, 0.10%; lipotropic factors: protein, 5% to 20%; methionine, 0.15%; choline, 0.05%.

Abundant evidence has shown that it is possible to induce fatty liver without adding cystine to the diet if choline and methionine are deficient; for example, the data for controls in Table 5 demonstrate this. However, adding cystine to the diets has advantages: (a) the production of fatty liver is assured; (b) the fat content of the diet can be very low making it possible to pellet the diet; (c) the shelf-life of the diet is increased; and (d) it is possible to allow quite high levels of choline avoiding the necessity of repeated extractions of the protein component with boiling ethanol.

After 1950, commercial diets became available and they contain high levels of cystine. The composition of a popular, amino acid-defined diet for inducing fatty liver in mice is as follows: methionine, 0%; choline, 0%; cystine 0.37%; corn oil, 5.0% (Diet # 518810, Dyets, Inc. Bethlehem, Pennsylvania). The corresponding control diet has the following composition: methionine, 0.17%; choline, 0.6%; cystine, 0.37%; corn oil, 5.0% (Diet # 518754).

Since diet # 518810 has no choline and no methionine, it would be hepatosteatogenic without adding any cystine. However, cystine is present at three to four times the hepatosteatogenic level and definitely in the toxic range. In the control diet #518754, choline is at least ten times the optimal level and more than sufficient to counteract the toxic effect of the cystine. Therefore, this system is unnecessarily complicated - superimposing the toxic effects of cystine on the fatty liver already caused by the deficiency of methionine and choline.

Fatty liver is the first stage in a pathological sequence which progresses with time to include inflammation, liver necrosis, cirrhosis, fibrosis, and increased incidence of cancer. Following the early work on fatty liver (particularly in the years from 1945 to 1970), numerous papers were published showing that high dietary intake of cystine (but not taurine or cysteic acid) caused these liver conditions as well as kidney damage; (this literature has never been reviewed, for an example see Earle [14]). A survey of these papers shows that the experimental diets had a cystine content of 0.5% or greater (as much as 6%) and had a cysteine/methionine ratio of above 5 as well as being choline deficient. The cystine content of these diets was 5 to 50-fold above the hepatosteatogenic level and in the toxic range. As with fatty liver, the other damages caused by cystine could be counteracted by appropriate amounts of choline (sometimes used at levels as high as 1% of the diet, e.g., Victor [15]). New insight into the mechanism of the relationship between cysteine and choline is discussed below.

### 1.4. The “Antilipotropic” Effect of Methionine

It had been accepted for twenty years that methionine is lipotropic when, in 1954, Harper *et al.* reported the surprising finding that, in diets with low protein, 0.3% to 1% methionine caused fatty liver [16] (Table 7). This effect of methionine which the authors called “antilipotropic” has been confirmed repeatedly [17] (for a partial bibliography, see [18]). In the early work, this effect was dependent on a low protein content of the diet but newer data suggest that the critical factor may be a low cystine content. Thus, a diet containing no cystine and 0.16% methionine (0.11% choline, 34% fat) did not cause fatty liver but the same diet in which methionine was increased to 1.14% caused severe fatty liver [19]. This “antilipotropic” effect of methionine in low protein or low cystine diets was difficult to explain until the condition of hyperhomoysteinemia was studied and found to be associated with fatty liver. It is now well-known that methionine loading by mouth causes a sharp increase in blood homocysteine. This shifted the blame to homocysteine as discussed below.

**Table 7:** Antilipotropic Effect of Methionine (from [16]).

Diet	Exp.	Methionine (% of Diet)	Liver (% Fat)
Casein 9% Corn oil 5% Choline 0.15%	1	0	3.7
		0.3	11.0
	2	0	4.2
		0.3	9.7
	3	0	4.8
		0.1	7.8
		0.2	8.3
		0.6	7.5

## 2. Hyperhomocysteinemia (HHE)

Genetic defects in removing homocysteine cause it to accumulate with severe pathological effects, first seen in humans [20] and reproduced by genetic manipulation in mice [21]. The defective enzymes are involved in converting homocysteine to cystathionine or to methionine. The homocysteine concentration in blood increases from the normal value of 10 µM to as much as several hundred µM. The homocysteine is present in blood mainly as the mixed disulfide with cysteine or protein sulfhydryl groups. Fatty liver is associated with this genetic defect both in humans [20] and experimental animals [21] along with disturbances in arteries, connective tissue, and the brain. Several factors have been studied as possible explanations of the connection between homocysteine and lipid metabolism. HHE is associated with marked increase in the activity of three enzymes associated with lipid synthesis: acetyl-Co A carboxylase activity increased 5 to 8-fold and fatty acid synthase activity increased 4 to 6-fold in livers of B_12_–deficient rats [22], and hydroxymethylglutaryl (HMG)-coenzyme A reductase activity increased 2-fold when liver cells were incubated *in vitro* with homocysteine [23]. The increase in HMGCoA reductase corroborates the effect of methionine in increasing the cholesterol fraction in the liver fat (see Table 4). The possible relationship of homocysteine to fatty liver via sulfane sulfur is discussed below.

## 3. Sulfane Sulfur

Sulfane sulfur is elemental sulfur with 6 valence electrons represented as S^o^ [24]. It readily accepts two dative electrons to complete the Lewis 8-electron rule and it has a high affinity for free electron pairs on other sulfur atoms. It attaches to sulfhydryl groups to give persulfides, R-SH + S^o^ → R-S-S-H; to alkyl sulfinates to give thiosulfonates, R-SO_2_^−^ + S^o^ → R-S(S)O_2_^−^; and to sulfite ion to give thiosulfate, SO_3_^2−^ + S^o^ → S_2_O_3_^2−^. The S-S bond in the products has been shown recently to be a 2-electron dative bond, not the 4-electron bond (S=S) as represented in the past [25,26]. The bond is weak [27] making the outer (sulfane) sulfur reactive. It transfers readily to other sulfur atoms and it is easily reduced from these structures by excess sulfhydryl compounds giving rise to hydrogen sulfide.

Sulfane sulfur has dramatic effects in increasing the health, vigor, viability, and replication of many cells types in numerous tissues of the body. Its protective effects are seen in three fields of research. (a) In cell cultures of immune systems, sources of S^o^ are highly stimulatory and S^o^ is required absolutely (at nanomolar concentrations) for *in vitro* proliferation of cells defective in the enzymes cystathionine gamma lyase (CGL) and methylthioadenosine phosphorylase (MTAP) [28]; (b) Garlic contains compounds which contain S^o^ or give rise to it and are associated with a remarkable list of beneficial health effects (reviewed in [29]); (c) Solutions of sodium hydrogensulfide (NaHS) which contain S^o^ in the form of polysulfide have been shown to have numerous protective effects [30]. Sulfane sulfur has at least three functions which may explain its beneficial effects. (a) It is involved in synthesis of anti-oxidant molecules such as iron-sulfur enzymes, superoxide dismutase, and glutathione; (b) It modifies tRNA whereby it may influence translation; (c) It binds to the sulfhydryl groups of many enzymes altering the activity of the enzyme [24,31]. There are highly-conserved carrier proteins which transport S^o^ to specific sites [31].

Homocysteine can give rise to S^o^ by the two direct mechanisms: (a) Homocysteine is deaminated to 2-keto-4-mercaptobutyrate (KMB) either by amino acid oxidase [32,33] or by transaminase [33]. The C-S bond is lablized by keto enol tautomerization of the gamma carbonyl group (Equation (1)) as studied by Nicolet [34]. H_2_S was detected in these systems [32,33]. In *in vitro* systems, the labile sulfur may be reduced to H_2_S by the excess substrate whereas, *in vivo*, it is transferred to a carrier protein.

COOH-CH(NH_2_)-CH_2_-CH_2_-SH → COOH-C(O)-CH_2_-CH_2_-SH → COOH-C(OH)=CH-CH-SH (1)

(b) Bacterial (and probably mammalian) CGL catalyzes degradation of cysteine-homocysteine mixed disulfide (cy-S-S-hcy) by gamma elimination giving rise to cysteine persulfide:

cy-S-S-hcy + H_2_O → NH_3_ + α-ketobutyrate + cy-S-SH (2)

This mixed disulfide, cy-S-S-hcy occurs in normal human blood [35] and its concentration in blood increases in parallel with homocysteine in HHE [36,37].

Homocysteine is converted to cysteine by transsulfuration and cysteine can give rise to S° by (a) transamination/deamination to give mercaptopyruvate in which the C-S bond is labilized by the beta carbonyl group, or (b) by beta elimination of cysteine disulfides catalyzed by several enzymes called “cysteine S-conjugate lyases”. (For full details and literature references on these systems, see another paper in this series).

## 4. New Findings on the Effects of Sulfur on Fatty Liver

### 4.1. New Findings

Two recent publications throw new light on the sulfur involvement in diet-induced fatty liver, one by Maclean *et al.* from the University of Colorado [38] and the other by Luo *et al.* General Hospital of Chengdu, China [39]. Both studies use methionine-choline deficient (MCD) diets to produce fatty livers. The first demonstrates a lipotropic effect related to homocysteine metabolism and the second relates it to sodium hydrosulfide. In the first study, the mouse gene for CBS was inactivated and an introduced human CBS gene was expressed at a low level causing plasma homocysteine to accumulate to ~200 µM (normal value ~ 5 µM). These mice did not develop fatty livers spontaneously. Nor did they develop fatty liver when fed the MCD diet #518810 as did the wild type mice. Further, they were protected from the hepatotoxic effect of tunicamycin in comparison to wild type mice. A possible explanation is that the protective effect is related to the elevated homocysteine and its conversion to S^o^; however, the authors attributed the effect to cystathionine which is also increased in the blood plasma of these mice to ~8 µM from a wild type value of ~2 µM.

In the second paper, genetically-normal rats were fed MCD diets and some were injected daily intraperitoneally with solutions of NaHS delivering the equivalent of 3.0 mg of sulfur atom per day. By comparison, these rats received the same amount of sulfur as rats eating 10 gm per day of a diet containing 0.15% methionine. The sham-treated rats developed fatty liver but those receiving NaHS did not. A protective effect of NaHS in fatty liver is not surprising. Since 1996, solutions of NaHS have been shown to have protective effects against tissue damage in a large number of studies (for a comprehensive review see [30]). The damaging effect has frequently been induced by ischemia followed by reperfusion or by toxic chemicals. The tissues shown to be protected by NaHS include liver as well as kidney, heart, lung, colon, and endothelium. It has been pointed out that the solutions of NaHS contain significant amounts of sulfane sulfur as polysulfide, Na-S_n_-SH, and that the effects of NaHS are probably attributable to the sulfane sulfur rather than the sulfide [31,40].

### 4.2. The Lipotropic Effect of Methionine Explained

The lipotropic effect of methionine has been traditionally attributed to its provision of methyl groups via S-adenosyl methionine for the conversion of phosphatidylethanolamine to lecithin with subsequent export of lipoproteins from the liver. The new data suggest an additional effect of methionine as a source of sulfane sulfur which then maintains a normal metabolic balance in liver cells under adverse conditions. This mechanism was first suggested by the mouse model of Maclean *et al.*, in which high levels of homocyteine (200 uM) could give rise to S^o^ and this appears to be confirmed in the second paper where solutions of NaHS would provide the S^o^.

### 4.3. The Hepatosteatogenic Effect of Cystine Explained

**Scheme 1:**
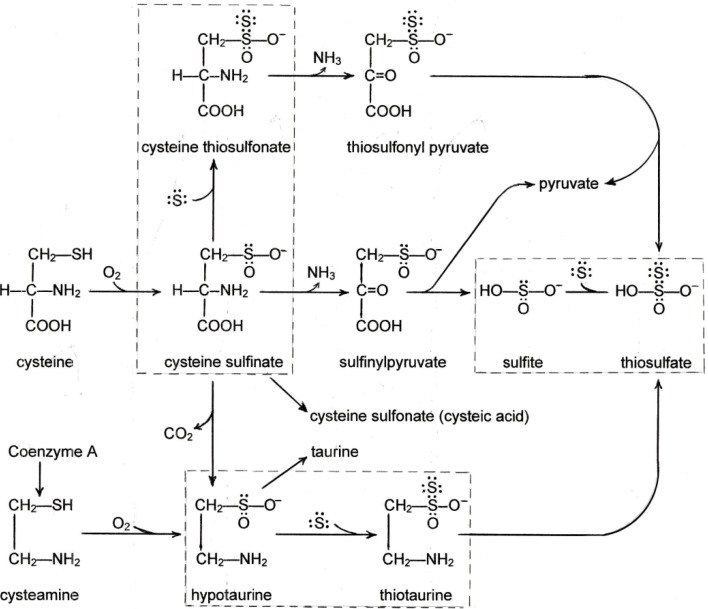
Oxidative Metabolism of Cysteine and Cysteamine.

The oxidative catabolism of cysteine and cysteamine is shown in Scheme 1. Both cysteine and cysteamine undergo oxygenation by separate dioxygenases to yield cysteine sulfinate and hypotaurine respectively. The cysteine sulfinate can be decarboxylated to hypotaurine or deaminated to sulfinyl pyruvate which is unstable and spontaneously releases sulfite. In this series there are three compounds derived from cysteine which have an unshared electron pair that attracts sulfane sulfur. (a) Cysteine sulfinate adds S^o^ to become cysteine thiosulfonate [41] which has been identified in the urine of rats fed extra cystine [42]; (b) Hypotaurine adds S^o^ to become thiotaurine [43] which also is excreted in the urine when rats are fed extra cystine [44]; (c) Sulfite ion adds S^o^ to become thiosulfate [45] which is excreted in normal urine. In the rare genetic defect in sulfite oxidase (which catalyzes the oxidation of sulfite to sulfate), thiosulfate excretion in the urine is increased 30-fold along with some sulfite (normally undetectable) [46], confirming the S^o^-binding role of sulfite. Cysteine thiosulfonate and thiotaurine can be deaminated resulting in the release of thiosulfate [47]. The dioxygenase system is tightly controlled and increases in response to increased cystine intake [48]. Therefore, diets high in cystine would cause increased flow of S^o^-binding intermediates which could deplete this essential form of sulfur in the body. If S^o^ is required to prevent fat accumulation in the liver, as discussed above in the consideration of methionine, conversely its removal by cystine catabolites would promote fatty liver.

### 4.4. The Relationship between Cystine and Choline

There is a large body of evidence that excess intake of cystine causes damage to the liver, kidneys, and pancreas and that choline, at sufficient amounts, prevents these effects even when cystine is ingested in amounts up to 5% of the diet. Therefore, there is a powerful metabolic relationship between cystine and choline which has never been explained. In light of new findings, it may be possible to explain this relationship on the basis of three-well established tenets. (a) Cysteine metabolism in the liver gives rise to bisulfite ion as discussed above; (b) Also in the liver, choline dehydrogenase converts choline to betaine aldehyde which is free in the tissue before further dehydrogenation to betaine; (c) A characteristic reaction of aldehydes is the spontaneous addition of bisulfite. Betaine aldehyde is known to undergo this facile addition reaction [49]:

(CH_3_)_3_N^+^-CH_2_-C(O)-H + HSO_3_**^−^** → (CH_3_)_3_N^+^-CH_2_-CH(OH)-SO_3_**^–^** (3)

This spontaneous reaction removes the sulfite derived from cysteine catabolism and prevents the sulfite from trapping and depleting S^o^. This relationship is summarized below in Conclusions.

The above considerations suggest that fatty liver might be induced by sulfite which is used as a preservative in many foods or by the elevated sulfite which occurs in sulfite oxidase deficiency [46]. However, just as cystine enhances hepatosteatogenesis only when methionine and/or choline are deficient, the same is probably true for exogenous sulfite. Indeed, a survey of the literature on sulfite toxicity testing and on sulfite oxidase deficiency shows that fatty liver is not mentioned. However, sulfur dioxide administered to mice by inhalation has been reported to cause fatty degeneration of the liver [50]. (Sulfur dioxide forms bisulfite ion when in contact with water at physiological pH.)

### 4.5. The Antilipotropic Effect, the Double-Edged Sword

Just as methionine was found to be lipotropic when present in the diet at low amounts and antilipotropic at high amounts, the end-product, sulfane sulfur, has two opposing effects. The findings from the new reports indicate that at appropriate low concentrations, sulfane sulfur prevents fatty liver. Conversely, when its precursor homocysteine is present at very high blood concentrations as in HHE, sulfane sulfur could be formed in excessive amounts and cause fatty liver. The hepatosteatogenic mechanism of S^o^ is not clear at present. As cited above, three enzymes of lipid synthesis are increased in HHE. This could be caused by S^o^ acting at the translation level via tRNA or it could be due to its binding to the enzymes and activating them. It is likely that other unidentified enzymes are also altered in activity in this context.

Similar concentration-dependent and opposite effects of sulfane sulfur have been documented before. In cultures of cells defective in two enzymes of sulfur metabolism (CGL and MTAP), sulfane sulfur is absolutely required for cell proliferation but it has a very narrow effective concentration range at the nanomolar level [28]. When its concentration exceeds the optimal level, the cells die. The first and characteristic sign of toxicity is the presence of multi-nucleated giant cells with as many as 8 nuclei per cell [24]. In this regard, it is interesting that multinucleated cells are seen in the fatty livers of humans with CBS defect [20], the CBS knock-out mouse [21], pups from B_12_-deficient rats [3], and in the bone-marrows of humans with pernicious anemia. If multinucleation is taken as a marker of excessive levels of S^o^, these findings suggest that the harmful effects of excessive homocysteine are due to the generation of toxic levels of S^o^.

### 4.6. Health Implications

Fatty liver is related to several medical conditions currently recognized as “epidemic”. These include non-alcoholic fatty liver disease, obesity, metabolic syndrome, and type 2 diabetes. The causation of these conditions can involve many factors aside from diet including lack of exercise, genetic predisposition to insulin resistance, dysfunction of other hormones (leptin, adiponectic), impaired beta oxidation, hepatotoxins, and some viruses [5]. Within the “diet” category, there are several factors which contribute to fatty liver such as high caloric intake, high fat intake, and imbalance of essential amino acids or essential fatty acids. However, among all of these factors, the most immediate and potent factor in promoting fat accumulation in the liver appears to be an imbalance of dietary sulfur amino acids. The new findings relate this effect of sulfur amino acids to sulfane sulfur. This brings fatty liver into the fold with several other disorders in which sulfane sulfur has been reported to have beneficial effects in the fields of garlic research and “hydrogen sulfide” (neurodegenerative diseases, carcinogenesis, atherosclerosis, type 1 diabetes, recovery from oxygen deprivation, and others). The diversity of the effects of sulfane sulfur is consistent with it having generalized functions. These functions appear to be tissue non-specific such as metabolic regulation (either at the translation level via tRNA or at the enzyme level) or maintenance of redox regulators (iron-sulfur cluster, superoxide dismutase, glutathione).

This raises the question of possible benefits from increasing the intake of precursors of sulfane sulfur and, indeed, there is rapidly growing evidence for such benefits. In this regard, the most potent sources of S^o^ are the xenobiotics first used in cultures of immune systems, mercaptoethanol disulfide and cystamine. Mercaptoethanol disulfide yields S^o^ when dehydrogenated by alcohol dehydrogenase [28]. When fed life-long to mice, mercaptoethanol dramatically prevented obesity and lowered serum cholesterol [51]. In other studies, mercaptoethanol totally prevented the cancers that normally kill mice [52] and caused a 50% increase in longevity [51,53,54]. Cystamine gives S^o^ when metabolized by diamine oxidase [28]. Cystamine has potent anti-HIV effects when the virus is cultured on lymphocytes [55]. Cyst(e)amine (sometimes used in the reduced from but undoubtedly containing significant amounts of the disulfide in an aerobic environment) has anti-cancer effects (see [56] for extensive literature) and it is in clinical trial for neurodegenerative diseases [57]. Thiotaurine (which contains S°) is excreted in the urine when excess cystine is ingested by rats [44] but it can also serve as a source of S° through the mobilizing effect of glutathione (GSH) through the formation of the persulfide (GSSH) [58]. Treatment with thiotaurine has been shown to correct numerous stigmata of streptozacin-induced diabetes in rats (see [59] for key to literature) and to greatly increase the viability of isolated human neutrophils [58].

Natural sources of sulfane sulfur include any of the alliums (e.g., garlic, onion, chives), the brassicas (e.g., cabbage, broccoli, cauliflower), asparagus, Shitake mushrooms (containing lenthionine), and truffles (containing dimethyldisulfide). The old-fashioned “spring tonic” was composed of equal parts of sublimed sulfur and blackstrap molasses. There are no data on how much of the sulfur is absorbed from this tonic but it is not likely to be much or the recipients would be poisoned. However, possible benefits from low chronic intake of elemental sulfur or thiosulfate (both containing sulfane sulfur) should be investigated.

There is a further health implication from the evidence reviewed here. Data cited above show that there needs to be a balanced dietary intake of cystine and methionine and that excessive or imbalanced intake of these sulfur amino acids can promote liver pathology. The proper balance is inherent in the normal diet. However, methionine and cystine products are available at health food stores. Excessive use of one or other of these could have harmful effects. Again, this would be more likely on a background of choline deficiency which, according to choline expert, S. H. Zeisel, is not uncommon [60].

## 5. Conclusions

**Scheme 2:**
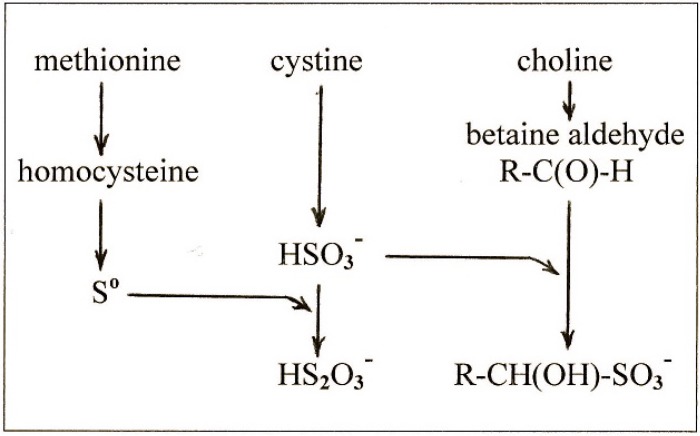
Metabolic Relationship between Methionine, Cystine, and Choline.

The study of diet-induced fatty liver has revealed a complex relationship of the sulfur amino acids and choline to fat metabolism in the liver. Many aspects of this relationship have been difficult or impossible to explain in the past. Recent findings implicating S^o^ provide a new interpretation of these relationships as summarized in Scheme 2. This interpretation proposes that there are three reactions by which sulfur amino acids and choline influence the fat content of the liver:

- methionine gives rise to S^o^ which is the ultimate lipotropic agent;
- excess cystine gives rise to excess sulfite which depletes S^o^ to suboptimal levels causing fatty liver;
- and choline gives rise to betaine aldehyde which removes the sulfite and blocks the depletion of S^o^.

Table 8 shows how this interpretation can be applied to published experimental data on fatty liver.

**Table 8:** Interpretation of the Effects of Sulfur Amino Acids and Choline on Fatty Liver. The causation and prevention of fatty liver are interpreted according to the rationale outlined in this paper. S^o^ represents sulfane sulfur bound to functional sites; SO_3_ represents bisulfite ion and other sulfinyl products which bind and remove S^o^; the word “choline” implies that it acts through its partial oxidation product, betaine aldehyde.

Condition Causing Fatty Liver	Fatty Liver Prevented by:	Ref.
Deficient methionine → low S^o^	Choline → decreased SO_3_→ increased S^o^	[9]
Deficient choline → high SO_3_→ low S^o^	Methionine → increased S^o^ production	[7]
Excess methionine → high S^o^	Cystine → increased SO_3_→ decreased S^o^	[16,19]
High cystine → high SO_3_→ low S^o^	Choline → decreased SO_3_→ increased S^o^	[9,10]
Same	Methionine → increased S^o^	[13]
High cystine, low methionine, low choline → high SO_3_→ low S^o^	Increased homocysteine → increased S^o^	[38]
Same	NaHS containing S^o^	[39]

The broad view can be summarized as follows. It is well-known that sulfur metabolism is a tightly regulated system [48]. The phenomenon of diet-induced fatty liver shows that this regulatory system is disrupted when the input of sulfur amino acids is imbalanced causing secondary disturbances in lipid metabolism. Until recently, it seems that a key factor in the regulatory process was missing. The recent findings implicating sulfane sulfur bring a new understanding of how sulfur metabolism may affect other metabolic systems through the formation of sulfane sulfur. This form of sulfur is just now being recognized as a versatile regulatory agent in many other systems and it remains to be discovered how it may control the physiological processes involving liver lipids. An understanding of this subject is particularly important as medical science addresses the life-style-induced health problems that are affecting the human society today.
